# West Nile Virus: An Update on Pathobiology, Epidemiology, Diagnostics, Control and “One Health” Implications

**DOI:** 10.3390/pathogens9070589

**Published:** 2020-07-19

**Authors:** Gervais Habarugira, Willy W. Suen, Jody Hobson-Peters, Roy A. Hall, Helle Bielefeldt-Ohmann

**Affiliations:** 1 School of Veterinary Science, University of Queensland Gatton Campus, Queensland, QLD 4343, Australia; g.habarugira@uq.net.au; 2 Australian Centre for Disease Preparedness, The Commonwealth Scientific and Industrial Research Organization, Geelong, VIC 3219, Australia; Willy.Suen@csiro.au; 3 School of Chemistry &Molecular Biosciences, The University of Queensland, St. Lucia, QLD 4072, Australia; j.peters2@uq.edu.au (J.H.-P.); roy.hall@uq.edu.au (R.A.H.); 4 Australian Infectious Diseases Research Centre, University of Queensland, St. Lucia, QLD 4072, Australia

**Keywords:** West Nile virus, pathogenesis, control, one health

## Abstract

West Nile virus (WNV) is an important zoonotic flavivirus responsible for mild fever to severe, lethal neuroinvasive disease in humans, horses, birds, and other wildlife species. Since its discovery, WNV has caused multiple human and animal disease outbreaks in all continents, except Antarctica. Infections are associated with economic losses, mainly due to the cost of treatment of infected patients, control programmes, and loss of animals and animal products. The pathogenesis of WNV has been extensively investigated in natural hosts as well as in several animal models, including rodents, lagomorphs, birds, and reptiles. However, most of the proposed pathogenesis hypotheses remain contentious, and much remains to be elucidated. At the same time, the unavailability of specific antiviral treatment or effective and safe vaccines contribute to the perpetuation of the disease and regular occurrence of outbreaks in both endemic and non-endemic areas. Moreover, globalisation and climate change are also important drivers of the emergence and re-emergence of the virus and disease. Here, we give an update of the pathobiology, epidemiology, diagnostics, control, and “One Health” implications of WNV infection and disease.

## 1. Introduction

West Nile Virus (WNV) is a zoonotic, mosquito-borne flavivirus, one of about 75 virus species of the *Flaviviridae* family [1]. WNV belongs to the Japanese encephalitis virus [2] serocomplex together with St. Louis encephalitis virus (SLEV), Murray Valley encephalitis virus (MVEV), and Alfuy virus (ALFV) [2,3]. It was first isolated in the West Nile Province of Uganda in 1937 from a febrile patient [4,5,6]. Initially, the virus was considered of less human importance as it only caused mild, subclinical infections [7]. However, the virus has been responsible for many cases of morbidity and mortalities in different animal species, including birds [8,9,10,11,12,13,14,15,16,17], horses [6,18,19], sheep [20], reptiles [21,22,23], cats [24], and rodents [6,25,26,27,28,29]. Over the last two decades, there have been notable increases in human and equine cases.

WNV is transmitted by a mosquito vector of the genus *Culex* through hematophagy [30,31,32]. However, some other ways of transmission, including ingestion, aerosol, and direct contact, have been reported in experimental settings [33,34], in humans (intrauterine and breastfeeding transmission) [35,36,37], and recently also in farmed alligators and crocodiles [38,39,40,41]. To date, only one case of vertical transmission has been reported in humans [36,42,43]. WNV transmission through blood transfusion and organ transplant have also been reported in humans [44,45]. The shedding of the virus in urine during the acute phase of infection also suggests that transmission through contact with environmentally contaminated material might be possible [46]. Despite much effort invested in vaccine development, there is currently no registered vaccine against WNV for use in humans.

There have been several reviews covering various aspects of WNV, including virus ecology and pathobiology [1,47,48,49,50], epidemiology [1,51,52,53], medicine and clinical pathology [1,44,52,54], and vaccine development [55,56,57]. This review provides a comprehensive update on WNV, focusing on virus biology and pathobiology, epidemiology, diagnostics, public and One Health importance and control, including new approaches made towards vaccine development, as well as other modes of prevention and treatment.

## 2. Virus Biology

### 2.1. Genetic Organisation and Virus Replication

**Figure 1:**
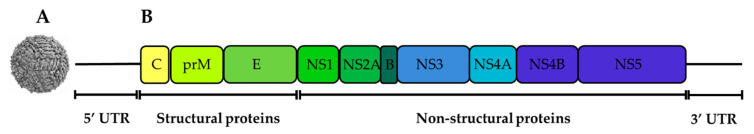
The structure of WNV virion (**A**) and 11 kb long viral genome represented with one ORF encoding 3 structural and 7 non-structural proteins (**B**) Source: adapted from De Filette et al. [74].

Like all flaviviruses, WNV contains a positive-sense, single-stranded RNA [ssRNA(+)] genome of approximately 11 kb. The genome is enclosed within an enveloped, icosahedral nucleocapsid with mature virions appearing spherical in morphology with an approximate diameter of 50 nm [58,59]. The viral genome contains a single open reading frame (ORF) coding for a polyprotein that is cleaved both co- and post-translationally [60]. The cleavage of the polyprotein is facilitated by both the host cell and the viral proteases and gives rise to structural and non-structural proteins [61]. The three structural proteins include the capsid (C), pre-membrane (prM), and the envelope (E) proteins [62] (Figure 1). There are seven non-structural (NS) proteins, encompassing NS1, NS2A, NS2B, NS3, NS4A, NS4B, and NS5, and they all play a crucial role in genome replication [63,64,65,66,67,68,69] (Figure 1; Table 1). The open reading frame is flanked on either side by 5′ and 3′ untranslated regions (UTRs), respectively [70,71,72]. The WNV genome has 96 nucleotides in the 5′ NCR and 632 nucleotides in the 3′ NCR with some variations between various strains [73]. Each of the viral proteins, either structural or non-structural, play a different and specific role in the biology and/or the pathogenesis of WNV infections (Table 1).

#### 2.1.1. The Capsid (C) Protein

The C protein is the WNV core protein and is made up of about 105 amino acid residues, most of which are charged and distributed across the protein [48,75], with some genetic variations occurring amongst WNV linages, strains, and isolates [76,77,78,79]. The C protein plays an important role in virus replication through its interaction with E3 ligases such as HDM2 [80]. The C protein also plays a role in the degradation of its binding proteins through the proteasome pathway [81,82,83]. The key role of the C protein is in nucleocapsid assembly; thus, the C protein shelters the viral genetic material (RNA). During viral replication, the RNA encapsidation and uncoating is enhanced by the binding of the C protein to the viral genomic RNA. It has been suggested that the encapsidation and uncoating are achieved through recruiting and releasing the viral genome [81,82]. Like in other flaviviruses, the WNV C protein is functionally flexible; hence, it can survive and adapt to severe or harmful mutations that would be fatal to other species of viruses [84]. The presence of the viral C protein in the host cell also plays a role in disease pathogenesis. The C protein induces cytotoxic effects in infected cells exhibiting cell cycle arrest in G2 phase [84,85]. Moreover, the C protein instigates the upregulation of caspase-9 and activation of the apoptosis pathway and subsequent cell death [85].

#### 2.1.2. The Envelope (E) Protein

The E protein is a transmembrane protein and has a protective role for other viral components by maintaining the envelope integrity [48,86]. The architecture of the E protein is conserved among different flavivirus species, including WNV [87]. The E protein has three domains: domain I (DI), DII, and DIII. They all are interconnected by a flexible, pH-dependent hinge region. At the surface of the virions (mature or immature), the three distinct domains are arranged in an antiparallel dimer [86,88]. It is the most immunogenic of the flaviviral proteins and due to its critical role in virus entry of the target cell, it is the principal target for most vaccines and curative drug designs mainly through immunotherapeutic approaches. The most potent neutralising antibodies to WNV have been mapped to EDIII [89]. The neutralising antibodies act either by inhibiting virion-cell attachment, endocytosis, or membrane fusion [48,86].

Like in many flaviviruses, the E protein of most WNV strains contains a conserved N-linked glycosylation site at the 154–156 amino acid position in DI [90,91]. However, some WNV strains contain no N-linked glycosylation site in E [90,92]. For example, the 1937 prototype strain of WNV [93] and the 1960 prototype isolate of the WNV Kunjin strain (WNV_KUN_) E protein were both shown to be unglycosylated [94,95,96]. There is a putative association between glycosylation of the E protein and neuro-invasiveness of WNV in various host species [94], although that has recently been disputed [96]. N-linked glycosylation is apparently not a requirement for WNV virulence in avian species [97].

#### 2.1.3. The prM/M Protein

The membrane (M) protein results from the cleavage of the glycosylated prM protein by the trans-Golgi resident enzyme furin. The ‘pr’ segment is secreted, while the M protein, with two membrane-spanning domains, forms part of the virion membrane. Exposed on the surface of the immature virion, prM is believed to play a critical role in preventing the premature fusion of the E proteins with the membrane of the host cell [94]. The cleavage of prM by furin is essential for the virus maturity [89].

#### 2.1.4. NS1 Protein

Non-structural protein 1 (NS1) has a molecular weight of approximately 46–55 kDA [98,99]. NS1 occurs as a dimer and is secreted as a soluble high-density lipoprotein hexamer of three more stable dimeric subunits. The dimeric form is critical for efficient virus replication [100]. In the infected cell, the NS1 is present both extracellularly [cell-membrane-associated (mNS1)] as well as intracellularly and its role varies according to the location. The extracellular form of NS1 plays a role in the regulation and evasion of the innate immune system through modulation of complement. The intracellular form is indirectly involved in the virus replication and maturation. It has been reported that NS1 enhances the attachment of the virus onto the endoplasmic reticulum and ensures the stability of the virus in the host cell [98,101,102]. NS1 protein is critical to WNV replication due to its ability to evade the host’s immune system through inhibition of complement activation and inhibition of TLR3 [103]. NS1 is actively secreted during WNV infection; thus, it is a useful serological marker. Moreover, NS1 is a potential diagnostic marker for differentiating infected from vaccinated animals [104] when vaccines used do not contain WNV-NS1 [105]. In addition, intracellular NS1 is a valuable target for immunohistochemistry applied to tissues collected at necropsy [106,107,108,109,110].

NS 1 prime (NS1’) is an extension of NS1 protein and has been reported in various flaviviruses including WNV, Japanese encephalitis virus [2] and dengue virus (DENV) [71,98]. NS1’ has a molecular weight of about 52–53 kDa. It has been hypothesised that the extension of NS1 is caused by the cleavage at an alternative site in NS2A due to the −1 programmed ribosomal frameshift slippage, downstream of the NS2A protein gene [98]. However, attempts to localise the cleavage site have all been in vain [111]. It has been demonstrated that NS1’ plays a key role in WNV neuroinvasiveness [98,112].

#### 2.1.5. NS2A Protein

The NS2A protein of the flaviviruses is a membrane-associated small molecule made up of 231 amino acids. This protein plays a key role in virus replication, virus assembly, and host immune modulation by disrupting the host’s interferon (IFN) response [113].

#### 2.1.6. NS2B Protein

NS2B is a small, hydrophobic protein, and an essential co-factor of NS3 to fulfil viral protease activity [114,115]. These two proteins are highly conserved across different flaviviruses of clinical interest and are essential to virus replication [48,67].

#### 2.1.7. NS3 Protein

NS3 is the second largest flaviviral protein after NS5 with a molecular weight of approximately 69 kDa. NS3 is multifunctional including a serine protease at the N-terminal end [116,117] and a RNA helicase at the C-terminal end [118,119,120]. The NS3 protease activity is dependent on NS2B as a co-factor [67]. The NS2B-NS3 protease is crucial for viral replication and cleaves the newly translated polyprotein at the junctions NS2A/NS2B, NS2B/NS3, NS3/NS4A, and NS4B/NS5 as well as internal sites within C, and NS4A [62]. The NS3 RNA helicase interaction with NTPase is essential for viral RNA replication and virion assembly [121]. Due to its multifunctional role in virus replication, NS3 has been suggested as a good target for antiviral drug development [114].

#### 2.1.8. NS4A Protein

NS4A is a small hydrophobic, non-conserved and solely a transmembrane protein that plays a role in the virus replication process through rearranging the viral membrane [67,122]. Moreover, NS4A-NS1 interaction is needed for viral RNA synthesis [62]. It also has been suggested that NS4A plays various roles during virus replication depending on where it is cleaved [119,123]. It has also been speculated that NS4A may play a role as cofactor regulating ATPase activity of the NS3 helicase [119]. NS4A protein is also associated with immune evasion [48].

#### 2.1.9. NS4B Protein

The NS4B protein plays a crucial role in immune evasion through inhibition of WNV interferon signalling [124]. In addition, the attenuation of WNV replication *in vivo* due to various mutations in NS4B suggests its role in virus replication [124]. Although there have not been strong evidence, it is believed that the interaction of NS1 and NS4B modulates WNV replication [125].

#### 2.1.10. NS5 Protein

The NS5 protein is the largest and most conserved among the non-structural proteins (approximately 96 kDa). Like most flaviviruses, the WNV NS5 protein is comprised of a N-terminal methyltransferase (MTase) and a C-terminal RNA-dependent RNA polymerase (RdRP) domains. The two enzymes play a crucial role in virus replication [126,127]. During viral replication process, the NS5 MTase domain is involved in RNA capping [127]. NS5 is also an IFN-α and β antagonist, hence a virulence determinant via evasion of the innate immune response. It also inhibits the translation of IFN stimulated genes (ISGs) [67,128,129,130,131].

**Table 1:** Summary of WNV proteins and their function.

Viral Protein	Position in the Genome (Nucleotides)	Main Role	References
C	97-465	- RNA encapsidation and uncoating - Activation of apoptosis pathway and cell death	[62,81,82,85,132]
prM/M	466-741-742-966	- Virion assembly - Virus—host cell fusion	[62,94,133]
E	967-2469	- Viral binding and entry to host cell receptors - Virus particle protection - Viral membrane—host cell fusion	[48,62,86]
NS1	2470-3525	- Viral RNA replication - Enhancement of the attachment of the virus onto the endoplasmic reticulum - Virus stability - Immune evasion (inhibition of complement activation)	[62,98,101,102,103]
NS2A	3526-4218	- Viral RNA replication and virions assembly - Immune evasion (disruption of IFN transcription)	[62,113,134]
NS2B	4219-4611	- Cofactor for NS3 protease activity - Virus replication and assembly	[48,62,67]
NS3	4612-6468	- Serine protease (N-terminal) - RNA helicase (C-terminal)	[48,62,118,119,120]
NS4A	6469-6915	- Viral membrane rearrangement - Inhibitor of interferon α/β host response	[62]
NS4B	6916-7680	- Immune evasion (inhibitor of interferon α/β host response) - Viral replication (enhancer of NS3hel activity)	[62,125,135]
NS5	768-10395	- IFN-α and β antagonist - Evasion of the innate immune response (IFN antagonist) - Methyltransferase (N-terminal), RNA-dependent RNA polymerase (C-terminal)	[62,67,128,129,130,131]

### 2.2. The Life Cycle of WNV

**Figure 2:**
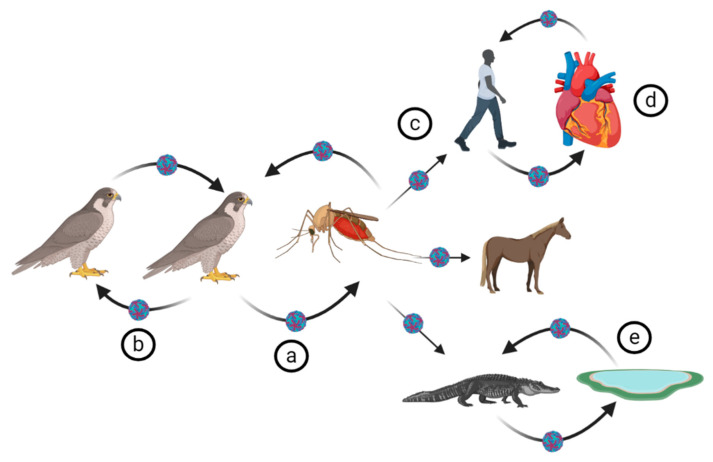
WNV lifecycle and transmission. (**a**) WNV maintenance between birds (reservoir) and competent mosquito vector, (**b**) WNV transmission via direct between birds in commercial farm setting, (**c**) WNV transmission to various hosts (human, horse and crocodile) via mosquito bite, (**d**) WNV transmission via blood transfusion and organ transplant in human, (**e**) WNV infection in crocodile through WNV contaminated water.

The WNV life cycle involves virus reservoirs (mainly birds, which can harbour the virus without signs of clinical disease), mosquito vectors (which also support viral replication), as well as final or incidental hosts. The latter are mainly infected during a mosquito blood meal, if the mosquito saliva titre exceeds 10^4^ TCID_50_/mL and may then develop clinical disease [42] (Figure 2). Final hosts are generally dead-end-hosts, except for the crocodilians which, unlike other final hosts, also amplify the virus [39,41]. Competent mosquito vectors acquire the virus from a viraemic vertebrate host during their blood meal. Following ingestion of the blood meal, the WNV reaches the mosquito midgut where the virus is amplified and spreads to the salivary glands prior to infecting the final host during the mosquito’s subsequent blood meal [136,137]. It was initially thought that, in the mosquito, the virus replication is strictly limited to the midgut. This was hypothesised based on failure to detect the virus out of peritrophic matrix barriers made of chitin and various other proteins [138]. However, WNV-NS1 has also been detected by immunohistochemistry in salivary glands, neurons in the ganglia, and eye cells in addition to the midgut tissues [137,139]. Interestingly, as is the case for other flaviviruses, WNV does not cause apparent disease in the mosquitoes [138]. After replication in the midgut, and other tissues, the virus starts a retrograde journey to the mosquito salivary glands via hemolymph. In the mosquito salivary glands, virus particles aggregate pending the mosquito’s feeding on a definitive host [136,140].

WNV vertebrate hosts, including reservoirs and incidental hosts, are infected during the uptake of a blood-meal by a WNV-infected mosquito. Mosquitos probe their blood vessels by injecting its saliva prior to sucking its blood meal. In addition to anticoagulation properties, the injected saliva contains proteins that interfere with the host’s T cell response, hence, initial cell mediated immune evasion and virus spread [141].

The virus infects the vertebrate host cell via cell receptor mediated endocytosis following cell-virus fusion [142]. Although other receptors, such as the mannose receptor and several glycosaminoglycans, have been suggested, dendritic cell-specific intercellular adhesion molecule 3-grabbing non-integrin receptor (DC-SIGNR) has been shown to be the main mediator for WNV cell entry [142,143,144]. Once the virus gets into the host cell endosomal vesicles, the viral E protein acidifies, triggering conformational changes and the viral and cellular membranes fuse [142]. Optimal viral membrane and cell endosomes/liposomes fusion is achieved at pH 6.3–6.9. After the fusion is optimally achieved, the nucleocapsid and viral RNA are released into host cell cytoplasm to initiate replication [145]. After replication in the cytoplasm of the infected cell, new virus particles acquire a lipid envelope by budding into the lumen of the ER and are matured via cleavage of prM (removal of pr by furin) during exocytosis and release from the cell.

It has been speculated that the DC-SIGN receptor is a key factor during vector-dependent infection of cutaneous macrophage and dendritic cells, although the subsequent virus spread within infected host could be DC-SIGN independent [142].

## 3. Genetic Diversification within the WNV Species

**Figure 3:**
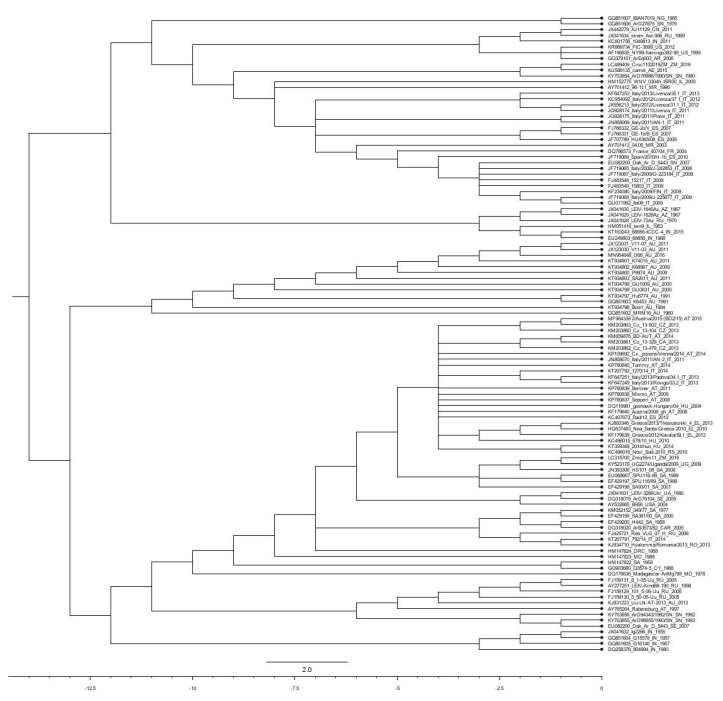
Maximum-likelihood phylogenetic tree of estimating the relationships of selected West Nile virus isolates. The tree was constructed with MEGA-X software version 10.1.8. The optimal tree was obtained using Maximum likelihood method, Nearest Neighbour Interchange (NNI) inference method. The phylogeny was tested with bootstrap replicates method (N = 1000). The evolutionally distances were calculated with the general time reversible (GTR) model, uniform rates. The tree was edited with FigTree v1.4.3 software (http://tree.bio.ed.ac.uk/software/figtree/). The scale represents at the bottom represent divergence time in millions of years ago (MYA). Each sequence used was labelled by GenBank accession number_isolate/strain name_country of isolation_year of isolation.

Based on biology, evolution, pathogenicity, and geographic distribution, WNV has been grouped into nine lineages [146,147,148,149,150,151] (Figure 3). Except for Koutango virus, the only member of lineage 7, WNV strains of lineage 1 and 2 are the most virulent and have been responsible for several outbreaks with severe neurological disease worldwide [152]. Lineage 1 is subdivided into 3 sub-lineages, including sub-lineage 1a encompassing the African, European, and Middle Eastern isolates [148,153]. The sub-lineage 1b comprises WNV_KUN_ strains from Australasia and the sub-lineage 1c, also known as lineage 5, comprises virus isolates from India [146,154,155].

Lineage 2, also neurotropic but with lesser virulence, comprises isolates from Sub-Saharan Africa, Madagascar, and Europe. Viruses in this lineage have caused several outbreaks in humans, horses, and birds [76,146,150,156,157,158,159,160].

Lineage 3 and lineage 4 comprise one virus isolate each. Lineage 3, also known as Rabensburg virus, has only one strain from the Czech Republic [161,162]. Lineage 4 consists of one WNV strain (LEIV-Vlg99-27889-human, LEIV-Vlg00-27924-human, Ast99-90 I-human) isolated from Russia [163,164]. A putative lineage 6 has been suggested based on NS5 gene sequence in WNV isolated from Spain (HU2925/06) [165].

Lineage 7 consists of Koutango virus (Flavivirus), first isolated in 1968 in Koutango, Senegal, and later in Somalia [166]. The Koutango virus was initially classified as an independent flavivirus species but later classified as a WNV strain [167,168]. Koutango virus was demonstrated to be more pathogenic than other virulent strains [107]. A putative lineage 8 virus was isolated from *Culex perfuscus* in Kedougou, Senegal [169].

The WNV-Uu-LN-AT-2013 strain, isolated in Austria, was proposed to form lineage 9 or to be part of lineage 4 as sub-lineage 4c. Although it was concluded that this isolate is not insect or mosquito specific, there are no reports about its *in vivo* pathogenicity either in humans or animals [151].

## 4. WNV Ecology

This section describes the virus biology including vectors and transmission, reservoirs, and host interactions.

### 4.1. Virus Transmission

WNV is primarily transmitted biologically by competent mosquitoes. Mosquitoes not only play a vectorial role but are also intermediate hosts, with some level of virus amplification prior to infecting definitive hosts [42,170,171,172]. WNV can also be nosocomially acquired mainly via organ transplants, needlestick, haemodialysis, and blood transfusion in humans. These modes of transmission were first reported during the first WNV outbreak in the USA [173,174,175,176,177,178]. Oral-faecal route of transmission has also been confirmed in American alligators and saltwater crocodiles [39,40,41,179]. Contact transmission in commercial geese farming has also been documented. It was thought to be generally associated with cannibalism and feather picking of infected birds [180]. There has been one reported case of WNV transplacental transmission in human [36]. Plausible breastfeeding transmission of WNV has also been documented [37]. Aerosol transmission among animal handlers and laboratory workers has also been hypothesised [174]. Behavioural risk factors have also been documented. A study by Lindsey et al. [181] reported alcohol abuse as a major risk factor for WNV infection and disease.

Blood transfusions and organ transplants from previously infected individuals are other sources of WNV infection [182]. WNV has been diagnosed in people who received whole blood as well as blood components including red blood cells, plasma, and platelets [45,177,183,184]. It has been shown that the virus might be present and viable in solid organs despite negative serology results. Thus, solid organ transplants pose a potential risk to recipients [44,173,182,183,185,186,187,188,189].

### 4.2. Biological Vectors of WNV

*Culex* mosquitoes are reported to be the primary competent vectors of WNV. However, several other mosquito species have been suggested to be vectors, although their competency varies [190,191,192,193,194]. There are geographical variations in vectors of WNV across the globe. In Africa, where the virus was first isolated, *Cx. univittatus* is the most competent vector of WNV-transmission to humans [195,196,197]. Following WNV discovery, there have been several experimental transmission experiments in various mosquito species. The first successful experimental transmission was in 1942 in *Aedes albopictus*, thus, a potential competent vector [198] Several other experimental infections were reported in two mosquito species, *Culex pipiens* and *Cx. tritaeniorhynchus*, most abundant in Africa [197]. However, other mosquito species such as *Cx. antennatus*, *Cx. univittatus*, *Cx. theileri*, *Cx. neavei*, *Ae. caballus*, *Ae. circumluteolus*, *Coquillettidia* spp., *Cx. poicilipes*, *Ae. albocephalus*, *Cx. quinquefaciatus*, *Mansonia* spp., and *Cx. neavei* also play a significant role in the transmission of the virus to both humans and horses in different parts of Africa such as South Africa, Egypt, Senegal, and Sudan [169,199,200]. *Culex interrogator* and *Cx. nigripalpus* were reported to transmit the virus in Mexico and other parts of Latin America [201]. When WNV was introduced into North America in 1999, two mosquito species, *Cx. restuans* and *Cx. salinarius*, were incriminated in the transmission of the virus [202]. However, later studies confirmed the role of other mosquitoes including *Ochlerotatus triseriatus*, *Ochlerotatus japonicus japonicus*, *Aedes albopictus,* and *Cx. pipiens* [203]. Further studies have detected WNV in about 150 mosquito species [204]; however, it was concluded that the key vectors of WNV in the USA are *Cx. pipiens*, *Cx. tarsalis*, and *Cx. quinquefasciatus* [205,206].

The main WNV vectors in Europe include *Cx. pipiens*, *Cx. modestus*, *Cx. molestus*, *Ochlerotatus caspius*, *Cx. torrentium*, *Anopheles maculipennis*, and *Coquillettidia richiardii* [198,207,208]. Culex annulirostris, a freshwater mosquito, is the main competent vector of WNV in Australia. The species is also the most laboratory competent vector [148,209]. However, WNV has been recovered from other mosquito species in Australia including *Aedes alternans*, *Ae. nomenensis*, *Ae. tremulus*, *Ae. vigilax*, *Cx. australicus*, *Cx. squamosus*, *Anopheles amictus*, and *Cx. quinquefasciatus*. None of the latter mosquitoes are as competent as *Cx. annulirostris* [209,210,211].

WNV is endemic in The Middle East countries, including Israel, Turkey, Jordan, Iran, and Lebanon. In that region, WNV is largely transmitted by *Cx. pipiens*, *Cx. perexiguus*, and *Ae. caspius* [212]. WNV vectors have also been documented in Asia, mainly in Pakistan and India where WNV is endemic. The main reported vectors are *Cx. vishnui* complex, *Cx. fatigans*, *Cx. tritaeniorhynchus*, *Cx. vishnui*, *Cx. bitaeniorhynchus* and *Cx. univittatus*, *Cx. pipiens fatigans*, *Ae. albopictus*, and *Cx. tritaeniorhyncus* [213].

WNV has been isolated from arthropods other than mosquitoes. These include hard ticks (*Hyalomma marginatum* and *Rhipicephalus sanguineus*), soft ticks (*Ornithodoros maritimus* and *Argas hermanni*), swallow bugs (*Oeciacus hirundinis*), and chicken mite (*Ornithonyssus sylviarum*) [198,214,215].

### 4.3. WNV Reservoirs

Several studies have demonstrated that various animal species such as Indian elephant (*Elephas maximus indicus*), Indian rhinoceros (*Rhinoceros unicornis*), ring-tailed lemur (*Lemur catta*), red panda (*Ailurus fulgens fulgens*), snow leopard (*Panthera uncia*), and babirusa (*Babyrousa babyrousa*) are susceptible to WNV infection [216,217,218]. However, it was concluded that only bird species can produce high enough virus titres to infect mosquitoes, which is a key requirement for the sustainability of the infection cycle. Birds not only play a role as reservoir but also are virus amplifiers and source of infection for dead-end-hosts [219]. Interest in researching the role of birds in the pathogenesis of WNV resulted from the detection of the virus in blood, spleen, and brain of pigeons from the Nile Delta in Egypt [217,220,221]. Subsequent susceptibility and permissiveness studies have been conducted in domestic and wild birds. Severe WNV disease has been diagnosed in chukar partridge (*Alectoris chukar*) [222,223], domestic geese (*Anser anser domesticus*) [222], domestic Impeyan pheasants (*Lophophorus impeyanus*) [223], Strigiformes (owls), Columbiformes (pigeons), Cathartidae (vultures), Corvidae (crows and related species), Gruidae (cranes), Pelicanidae (pelicans), turtle doves (*Streptopelia turtur*), bald eagle (*Haliaeetus leucocephalus*), a snowy owl (*Nyctea scandiaca*), flamingos (*Phoenicopterus* spp.), cormorants (*Phalacrocorax* spp.), American crows (*Corvus brachyrhynchos*), bald eagle (*Haliaeetus leucocephalus*), and cormorants (*Phalacrocorax* spp.) [15,218,224,225,226].

Although bird species are generally WNV reservoirs, investigations conducted during the WNV outbreak between 1999 and 2001 in the USA revealed *Corvus* species are the most susceptible to the diseases and the main amplifier [226,227,228,229]. Following the 1999 WNV outbreak in American alligators in the Americas and WNV associated “pix” lesions in saltwater crocodiles in Australia, experimental studies suggested that American alligators and saltwater crocodiles are also WNV amplifiers with high enough titres in their blood to potentially transmit the virus to mosquitoes [39,41].

Raccoons (*Procyon lotor*) were thought to be potential reservoirs and amplifiers of WNV in Europe but that hypothesis is still surrounded by controversies and requires more studies [230,231]. Seroprevalences studies of WNV in raccoons in the USA have reported WNV seroprevalence ranging between 34–54% [230,232,233]. Viremia and virus shedding profiles in experimentally infected Fox squirrels (*Sciurus niger*) suggested their ability in WNV infection maintenance and spread to final hosts [234].

## 5. Pathogenesis of WNV

**Figure 4:**
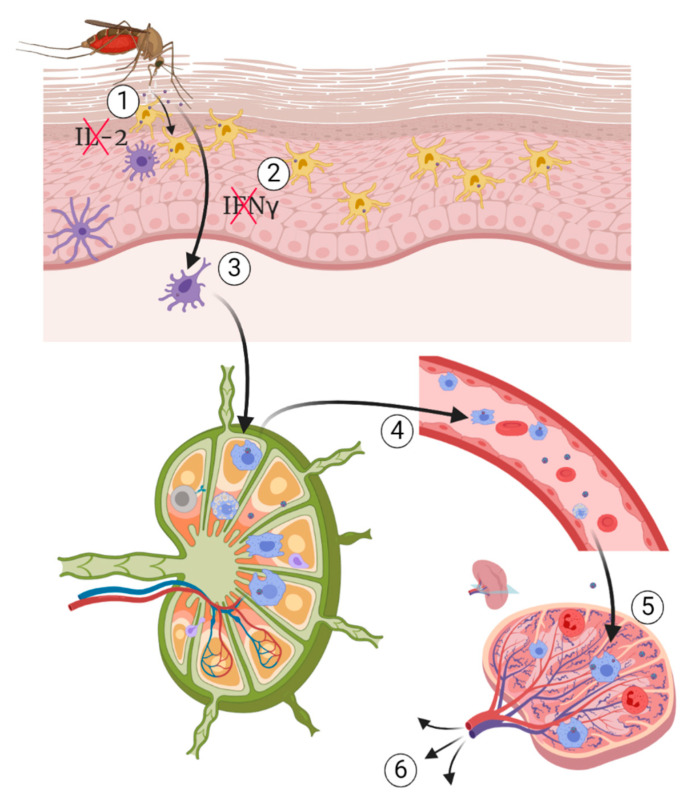
Pathogenesis of WNV infection. (**1**) *Culex quinquefasciatus* transmitting WNV during a blood meal on susceptible host and releasing its infectious saliva, (**2**) immunomodulation by mosquito’s saliva followed by infection of keratinocytes and Langerhans cells, (**3)** migration of infected cells to nearby draining lymph nodes, (**4**) viremia followed by migration of infected macrophage from the lymph nodes, and (**5**) spleen from which the virus spread to other organs of tropism. Source: Adapted from Petersen et al. [1].

The pathobiology of WNV infection in human and other mammalian, avian and reptilian species has been extensively studied. There is no single proven pathogenesis of WNV; however, some theories of the pathogenesis of WNV in mammals have been suggested. Following an infectious mosquito bite, the virus replicates locally at the injection site in the keratinocytes and Langerhans cells of the epidermis, a specialised type of dendritic cells associated with the skin [1,235]. The local virus replication is enhanced due to the immune modulation of the host response by the mosquito saliva through two mechanisms, including alteration of leukocyte proliferation and recruitment to the site of bite, and cytokine signalling by suppressing the production of interleukin (IL) 2 and IFNγ [141,236]. It is thought that dendritic cells could be among the early primary targets of WNV infection. This hypothesis was supported by the expression of DC-SIGN (also known as CD219) by dendritic cells during viral infection [144,237]. It has been hypothesised that infected Langerhans cells migrate to the draining lymph nodes in which the virus replicates further. Infected cells and free virus particles are picked up by macrophages and cleared either directly through phagocytosis or indirectly enhanced antigen presentation, cytokine, and chemokine secretion [238]. While macrophages clear the infection, virus replication continues in dendritic cells in the lymph nodes [237,239,240]. WNV replication *in vitro* in B and T lymphocytes suggests that these cells are potentially among the primary target during early stage of infection *in vivo* [241]. The infection of the cells of the immune system and virus replication in the cells is associated with immune modulation and appearance of primary clinical signs at the end of the incubation period, which is in the range of 2 to 14 days post infection [42]. From the lymph nodes, the virus is spread to peripheral organs hematogenously (Figure 4). In some hosts such as avian species, the virus has a wide range of tissue tropism and can replicate in nearly all the body systems [110,242,243,244].

Based on clinical presentation, there are several forms of WNV infection, including the neuroinvasive form previously reported in many hosts, the gastrointestinal form, hepatic form, pancreatic form, cardiovascular form, and a cutaneous form characterised by erythematous macules in humans and lymphohistiocytic-plasmacytic inflammation in crocodilians [60,229,232,233,234,235,236].

Presently, based on the clinical presentation and pathology, three main forms have been suggested and studied in various host species (animal models). These forms include the neuroinvasive form, the cutaneous form, and the gastrointestinal form [60,229,233,235,236,237]. The form and severity of WNV infection depend on several factors, including the WNV strain and lineage, the host species and intrinsic susceptibility, and the viral tropism as well as some extrinsic factors such as environment and coinfection [230,238,239,240]. Following WNV infection, a cascade of proinflammatory cytokines and other protein genes are upregulated as part of the innate immune response [108,110,241]. The activation and release of these chemicals are essential in the initiation and maintenance of inflammation in the control of viral infections, including WNV [74,145,242]. However, overexpression and continuous upregulation of inflammatory cytokine genes, may be detrimental in some viral infections including WNV, by enhancing the severity of infection and/or inflammation leading to death, chronic or permanent morbidity and or sequelae such as immunopathology. This is a phenomenon commonly observed with increased upregulation of cytokines such as IL-2, IL-6, IL-12, IL-17A, IFN- γ, IFN-γ induced protein 10 (IP-10), granulocyte-macrophage colony-stimulating factor (GM-CSF) and proinflammatory chemokine osteopontin (OP). The cytokines and chemokines may remain upregulated, even long after recovery from WNV infection [243,244,245,246,247].

### 5.1. Pathogenesis of Neuroinvasive Form

The neuroinvasive form of WNV infection is the most severe form of the disease. It occurs in about 1% of human and equine cases of WNV infection [235,247,248]. In humans as well as in equine species, this form of disease is characterised by syndromes of meningitis, encephalitis, and acute flaccid paralysis/poliomyelitis [249]. The lesions include granulocytic meningitis, lymphoplasmacytic-histiocytic perivascular cuffing, and lymphoplasmacytic meningo-encephalomyelitis. Similar pathology is reported in naturally and experimentally infected alligators [38,40,179]. Currently, there are no reports of the neuroinvasive form of WNV infection in other crocodilian species.

The lesions in infected birds with the neuroinvasive form are like those in other hosts. They include meningoencephalitis as characterised by lymphoplasmacytic-histiocytic perivascular cuffing, mild to diffuse gliosis and glial nodules. Rarely, multifocal necrosis (malacia) in the gray matter of the brain has been observed [243,250,251].

**Figure 5:**
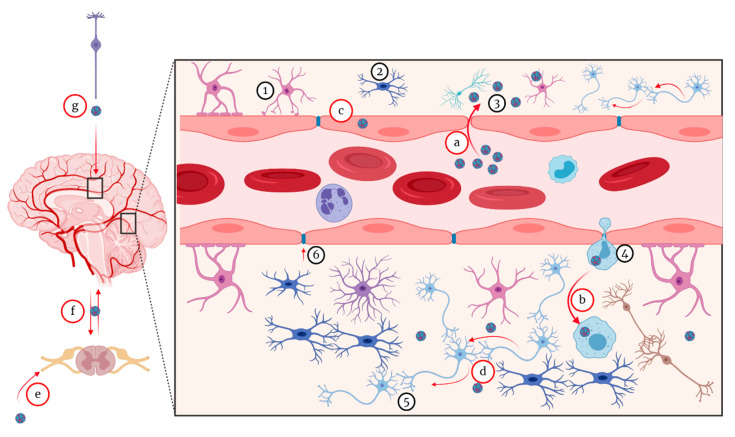
WNV neuroinvasive mechanisms. (**a**) Passive migration of free virus particles across the disrupted blood-brain barrier (BBB) through a “transudative” mechanism following increased vascular permeability, (**b**) “Trojan horse” mechanism through migration of infected macrophages into brain parenchyma, (**c**) direct infection of endothelial cell. (**d**) retrograde axonal transport of WNV, (**e**) WNV migration into spinal cord, (**f**) WNV migration from spinal cord to brain and vice versa, (**g**) neuroinvasive mechanism by transneural mechanism via olfactory nerve, (1) astrocyte, (**2**) microglia, (**3**) WNV particles, (4) transmigrating macrophage, (5) motor neuron, (6) blood-brain barrier (BBB) tight junction. Source: Adapted from Petersen et al. [1].

The mechanism of neuroinvasion by WNV has been debated over the years, with two scenarios still receiving foremost consideration. These include haematogenous and transneural routes of neuroinvasion (Figure 5). For both routes, several mechanisms have been proposed [252,253,254]. The first hypothesis consists of direct invasion of the CNS via a transendothelial mechanism following the infection of the endothelial cells. However, this mechanism may be host specific and even specific to some WNV strains [253,255]. It has also been demonstrated that brain endothelial cells are not the main target in the neuroinvasive form in the horse, human, or mouse [107,108,256]. Lim et al. [235] hypothesised that there is an association between levels and duration of viraemia and the WNV neuroinvasion. However, species susceptible to the neuroinvasive form, such as the horse, primates, and crocodilians, generally do not develop substantial and sustained viraemia. This therefore begs more questions regarding the link between viraemia and neuroinvasion, thus, requiring further studies.

#### 5.1.1. Hematogenous Route

Although haematogenous WNV spread has been suggested, it has not yet been definitively demonstrated *in vivo*; thus, this mechanism remains controversial. The active transportation by infected blood cells is the most plausible since the migration of infected cells begins before blood vessels leak [257]. However, passive haematogenous route following an increased vascular permeability during acute phase inflammation could be another possibility [258,259]. It is also plausible that neuroinvasion can initially occur by a non-haematogenous route initially, causing neuroinflammation, which then subsequently increased vascular permeability triggering haematogenous invasion. Given the complexity of cardiovascular anatomy and physiology, there is a need to elucidate the spreading mechanisms of WNV in animal models.

#### 5.1.2. Virus Passive Migration

Another hypothesis is the passive migration of free virus particles across the disrupted blood-brain barrier (BBB) through a “transudative” mechanism. This disruption of the BBB is a result of an increased vascular permeability due to the acute phase pro-inflammatory cytokines and chemokines [258,259,260,261,262]. However, this hypothesis has not yet been validated due to the lack of a universal *in vitro* or *in vivo* model. Moreover, there is another controversy about this hypothesis. Using a mouse model, JEV neuroinvasion was demonstrated to occur prior to the production of inflammatory cytokines and chemokines, which disrupted the BBB [263]. Similar observations were made in the WNV mouse model [260].

#### 5.1.3. The “Trojan Horse” Mechanism

The “Trojan horse” mechanism has also been hypothesised in several WNV studies and reviews [235,241]. It has been suggested that WNV neuroinvasion by the “Trojan Horse” mechanism is the result of the expression of lymphocyte and monocyte chemokines triggering the recruitment of infected peripheral leukocytes into the cerebral vasculature. The infected leukocytes then reach the brain parenchyma via the leaking BBB, resulting in appearance of neurological clinical signs and the development of lesions in the CNS [258,259]. Neuroinvasion by a “Trojan horse” mechanism was demonstrated for a pestivirus (of the Flaviviridae family), bovine viral diarrhea virus (BVDV), where, during transplacental infection of the fetus, infected microglial precursor cells, also known as amoeboid glial cells, brought the virus into the brain via the periventricular germinal zone from where it spread to differentiating neurons and glial cells [264]. However, because the innate immune response is still not fully developed in a mid-gestation fetus, this scenario may not be applicable to WNV infection of adult humans and animals, and more studies therefore should be conducted to elucidate this phenomenon.

#### 5.1.4. Transneuronal Mechanism

The transneural mechanism has also been proposed as another potential route of invasion of the brain by WNV. The transneural mechanism consists of virus migration following motor and sensory nerves from the point of entry. This mechanism is understudied as reflected in the scarcity of literature about this mechanism. Currently, two entry points have been suggested and these include from the peripheral somatic nerves into the CNS and from the olfactory nerve into the CNS [108,265,266]. It has been proposed that WNV spread from the point of entry to the central and peripheral nervous system by retrograde and anterograde axonal transport and/or via non-neural cells such as glial cells (astrocytes and microglial cells). By this mechanism, it has been suggested that the virus spread is mediated by viral release from distal axon to infect adjacent neurons leading to flaccid paralysis in some hosts when the sciatic nerve has been directly infected [265,267]. Flaccid limb paralysis is a result of neuronal injury associated with a severe infection and necrosis of the anterior horn neurons of the lumbosacral region (L5-S1) of the spinal cord [265].

Although several pathogenesis hypotheses have been proposed, none of them has been conclusive so far. The use of labelled infectious virus clones could potentially shed more light on different WNV pathogeneses, mainly the neuroinvasion mechanism.

### 5.2. Pathogenesis of the Cutaneous Form

Cutaneous manifestation of WNV infections have been extensively documented in humans. It is mainly characterised by an erythematous, maculopapular rash [268], and punctate exanthem affecting the extremities and the trunk [269]. Several reports have demonstrated the permissiveness of both keratinocytes, skin fibroblasts, and epithelial cells to WNV replication *in vitro* as well as *in vivo* [242,270,271,272,273,274,275,276,277,278]. The infection of skin epithelial cells is believed to be the cause of skin rash in 25–45% of the neuroinvasive cases in humans [271]. Viral antigen has been detected in the skin of naturally infected goshawks [278]. The replication of WNV in skin keratinocytes has been demonstrated at various stages of WNV infection and these are thought to be the primary target of WNV [271]. WNV replication was confirmed by immunohistochemistry (IHC) and virus titration at the subcutaneous injection site for up to 3 dpi [242]. Appler et al. [270] demonstrated the persistence of WNV in mouse skin for up to 14 dpi and WNV RNA for up to 30 dpi with or without clinical disease. Nevarez et al. [279], using RT-PCR confirmed the potential role of WNV in the development of lymphohistiocytic proliferative syndrome in farmed American alligators (*Alligator mississippiensis*). However, virus isolation was not successful; therefore, it could not be confirmed if the viral RNA detected was representing infectious or defective/inactivated virus. Similarly, WNV_KUN_ RNA was detected in skin lesions from farmed saltwater crocodiles (*Crocodylus porosus*) but live virus has so far not been isolated from these lesions [280]. In addition, WNV was successfully grown in a human skin primary cell line, the foreskin fibroblasts (HFF) [272,281]. Despite all the evidence of the susceptibility and permissiveness of skin cells, the pathogenesis of the cutaneous form of the WNV infection has not yet been elucidated.

### 5.3. Pathogenesis of the Gastrointestinal Form

WNV infection has been associated with gastrointestinal syndrome in humans as characterised by diarrhoea, enteritis, gastritis, pancreatitis, and hepatitis [282,283]. The gastrointestinal form of WNV infection has been reported in up to 30% of human cases [284,285,286,287]. However, to date, WNV replication in the human gastrointestinal tract (GIT) has not been investigated. Therefore, it would be worthwhile to test this either via WNV isolation, detection of viral antigen or viral RNA from faecal samples or GIT biopsy. The detection of viral antigen in the duodenal and proventricular enterocytes of naturally infected goshawks suggests replication of WNV could occur in the gastrointestinal tract of infected avian hosts [278]. Klenk et al. [39] demonstrated oral transmission of WNV in *Alligator mississippiensis* through feeding the latter with infected mice. WNV was isolated from cloacal samples from approximately 84% of the infected *A. mississippiensis*. The high virus titres (10^6.2^ PFU/swab) strongly suggested replication of the virus in the GIT. Alligators infected with WNV_NY99_ displayed a gastrointestinal clinical and pathological picture as characterised by inappetence, diarrhea, and bloating, preventing the affected animals from submerging into water [40]. Lesions in infected alligators include haemorrhagic enteritis, proliferative enteritis, ulcerative-proliferative esophagitis, fibrinous and necrotising colitis, histiocytic and necrotising hepatitis, and necrotising pancreatitis. WNV antigen positive cells were detected in the gastrointestinal tract of infected alligators by IHC [40,288]. However, these lesions are neither specific nor pathognomonic to WNV. Interestingly, although WNV genomic RNA was detected in both oral and cloacal swabs of saltwater crocodiles experimentally infected with WNV_KUN_ as well as in their un-infected pen-mates, *C. porosus* does not present with gastrointestinal clinical signs or pathological changes, and it remains unknown where exactly in the alimentary tract the virus is replicating in these animals [41].

### 5.4. Renal Form

The detection of WNV viral RNA in the urine of infected hamsters and humans raises concerns about a potential permissiveness of kidney cells and a renal form of the disease. Barzon et al. [46] detected WNV RNA from urine samples during the acute phase of infection in humans. In half of the investigated patients, viral RNA was detected in urine for much longer than in plasma. Moreover, the viral RNA load was higher in urine than in plasma. The renal form hypothesis is also supported by the detection of viral antigen by IHC in kidneys from patients who succumbed to the WNV infection [289,290,291]. WNV RNA was also detected in urine of infected patients for up to a month post infection [292]. The recovery of virus in urine from experimentally WNV infected hamsters for up to two months post recovery supports renal infection [293]. However, while hamster was a good animal model to investigate this form, the use of the WNV 385-99 strain, which has been adapted to hamster through serial passage of the virus isolated from urine, might have influenced these findings. Therefore, it would be worthwhile to investigate this form of the diseases in other animal model system such as rabbits and mice.

Although there is strong evidence for renal WNV infection in infected individuals, this form often exists in association with other forms of the disease, mainly the neuroinvasive form. Although the reasons for this association has not been elucidated, the immunocompromised status of the infected humans could be one explanation. Thus, the pathogenesis of the renal form is unclear, hence, to elucidate this, there is a need for modelling the infections in various laboratory animal models.

## 6. Clinical Presentation, Epidemiology, Infection Sequalae, and Persistence

### 6.1. Clinical Presentation and Epidemiology

There are several factors that influence the variation of clinical pictures of WNV infections. These factors include but are not limited to the host species, age, physiology status of the affected host, virus strain involved, virus tropism, and pathogenesis.

In humans, the clinical onset is marked by a high fever above 38 °C and headache. This initial clinical phase is also associated with depression, alteration of mental status with lethargy, and change of personality. Other signs include maculopapular rashes and erythematous petechial rashes. The meningoencephalitis form is characterised by nuchal rigidity, photophobia, flaccid paralytic presentation, and myasthenia. The gastrointestinal form is characterised by vomiting, nausea and anorexia [26,287,294,295,296,297,298,299,300,301]. Other signs include lymphadenopathy and hepatosplenomegaly [297]. Children, elderly individuals, and patients with chronic morbidities tend to develop a more severe disease [287,294,301].

Clinical signs in susceptible infected avian species include ruffled feathers, lethargy, ataxia, unusual posture, inability to fly or to hold head upright, head tremors, seizures, leg paralysis, nystagmus, and weight loss [160,302,303,304].

In reptilians, the clinical picture largely varies from subclinical in saltwater crocodiles to severe disease in American alligators. The two forms of the disease in the American alligators include the gastrointestinal form and a neurological form. Neurological signs include swimming in circles, ataxia, head and muscles tremors, head tilt, unbalanced swimming (sideway swimming, inability to submerge underwater), loss of leg control and neck spasms, and star gaze swimming [38,40,179]. Gastrointestinal signs include anorexia, bloating [40]. Aggression, weakness, cachexia, and immobility of the caudal part of the body are the main clinical signs in infected snakes [305].

Various risk factors have been identified and these include intrinsic and extrinsic risk factors. Age and gender are the main intrinsic predisposing factors to the disease. It has been reported that in humans older individuals are more susceptible than younger persons. The risk of acquiring the disease increases by 1.5 folds for every 10 years of age [306]. It has also been demonstrated that patients older than 75 years generally succumb to the infection [306], and this could be explained by ageing related innate immunoscenescence. Thus, ageing affects several antiviral pathways, including cellular pathways (macrophage related defences), cytokines such as type I IFN and TLR 3-mediated antiviral pathways leading to increased susceptibility to viral infection including WNV [307,308,309]. In addition, males are more at risk than females [310]. Pre-existing conditions and diseases such as cancers, cardiovascular diseases, and diabetes are also other major risk factors [44,306,311]. Immunosuppressed individuals have 40 times higher risk of contracting the disease and dying from WNV infection [185,187].

Other demographic risks factors for WNV infection have been documented. WNV infections outbreaks have been associated with urban settings in some countries such as Romania. People living in the basements of tall buildings were more at risk than the rest of the population. This has been linked to peri-domestic ecological behaviour of some mosquito vectors such as *Cx. pipiens-pipiens* and *Cx. Quinquefasciatus*, which are urban mosquitoes and commonly found indoors [312]. Moreover, reservoirs such as domestic fowl and scavenging birds play a big role in the spread of the virus and maintenance of the infection in urban settings [312]. In rural areas, WNV outbreaks, disease incidence, and prevalence have been linked with agricultural activities such as irrigation, which provides conducive conditions for mosquito breeding. Moreover, other agricultural activities such as bush clearing lead to the destruction of natural habitats for vectors and reservoirs, forcing them to flee to the proximity of human habitat [313].

Other risk factors include environment and climate. They both play an important role in the transmission dynamics of WNV. It has been demonstrated that spending more time outdoor or travelling to endemic regions pose a higher risk since individuals get exposed to mosquito bites [43,51,314]. This is common amongst outdoor professions and occupations. The main outdoor occupations at risk are farm workers, loggers, landscapers and groundskeepers, construction workers, painters, summer camp workers, and pavers, among others [315]. Soldiers and security guards are also at risk of contracting WNV considering the amount of time they spend doing outdoor activities [316,317,318].

Healthcare, laboratory workers, veterinarians, animal handlers, animal slaughterers, and butchers are also at higher risk of contracting WNV. These workers are exposed to WNV via needlesticks, accidental cuts, or contamination of open wound coming in contact with WNV infectious materials [315,318].

WNV is endemic is several parts of the world, including Africa, Middle East, Australia, Europe, and Asia. Currently, outbreaks have been reported in endemic areas as well as in non-endemic regions. Table 2 summarises the main WNV outbreaks that have been reported around the world.

### 6.2. Sequalae of WNV Infection

WNV infection is associated with lifelong sequelae ranging from physical morbidity to severe mental health issues. The most severe sequelae are generally observed in individuals who recover from the neuroinvasive form. Sequelae, including Parkinsonism tremors, poliomyelitis, meningitis, and cognitive disorders, have been documented in more than one-third of patients with confirmed infection [345].

The loss of hearing was also reported in individuals recovered from WNV infection. This sequalae is suggestive of the vestibular tropism of WNV, leading to chronic vestibulocochlear neuritis and loss of function [346]. WNV-infection of neurons in the spiral ganglion of the inner ear was detected in experimentally infected mice [108]. Long-term or permanent damage of other cranial nerves such as the ocular nerve has been reported for up to three years post recovery. Chronic retinopathy has also been reported [347]. Other reported sequalae included contracture of extremities, severe dysphonia, aphonia with complete unresponsiveness to commands, and with reduced sensation to pinpricking. Abnormal gates and movement disorders, loss of attention, and concentration have been linked to damage of the cerebral cortex. Anxiety and depression have also been reported among people recovered from WNV, particularly the neuroinvasive form [348,349,350,351,352].

Considering the severe sequalae of WNV infection, particularly in its neuroinvasive form, it would be interesting to investigate potential relationship between WNV infection and some debilitating neurological disorder such as Alzheimer’s and other types of dementia, multiple brain sclerosis, and Parkinson’s disease.

### 6.3. Persistence of WNV Infection

There is evidence of persistence of WNV infections in various host species, including mammals and birds with experimental or natural infection. An experimental WNV infection in hamsters demonstrated the persistent viruria for up to 52 days post infection. Furthermore, viral antigen was detected in epithelial cells in the kidneys [293]. Another experimental study demonstrated that WNV could persist in brain of infected hamsters for up to 89 days post infection, leading to neurological sequalae including neuron dysfunction and poliomyelitis-like syndrome [353]. In another experimental infection trial, infectious WNV was isolated from mice for up to four months post infection. Similarly, WNV RNA was detected from different mouse samples for up to six months post infection [270,354].

Interestingly, Pogodina et al. [355] also confirmed the persistence of WNV infection in monkeys experimentally infected via the intracranial route. In addition, the detection of WNV RNA from urine samples from 20% (n = 25) of patients for more than six years post infection strongly suggest the persistence of WNV is some patients [356]. Similarly, Baty et al. [292] confirmed the presence of WNV RNA in urine specimen several months post incubation in human.

Infection studies in house sparrows (*Passer domesticus*) also demonstrated WNV persistence. Infectious WNV was isolated from the host 12 weeks post infection and the viral RNA was detected for up to 18 weeks post infection [357,358,359].

The persistence of WNV in infected hosts beyond the viraemic period raises epidemiological and public health concerns. Further studies are needed to better understand the persistence mechanisms and their implication to both human and animal hosts.

### 6.4. WNV Surper-Infection

WNV reinfection has not been researched; thus, there are no reports of WNV reinfection either in clinical settings or under experimental conditions. However, it is believed that primary infection with WNV confers a life-long immunity; thus, infected and recovered persons cannot get a second WNV infection [360]. Papa et al. [361] reported the persistence of WNV IgM and IgG for up to three years post-infection in humans. Another study conducted in WVN seropositive blood donor volunteers reported neutralising antibodies in 94.4% (n = 54) for as long as five years post infection [362]. However, the results of this study should not be generalised given that the study sample involved individuals who voluntarily participated, thus, might not be truly representative of the population at large.

The persistence and increase of WNV IgG after natural infection suggest long-lasting immunity that could potentially confer protection to WNV reinfection. This aspect is clinically important and for better understanding, it should be modelled in various animal models.

### 6.5. Coinfections of WNV with Other Pathogens

Various pathogens can share hosts, and this phenomenon can occur through various mechanisms [363,364,365,366,367]. There are scenarios where an infection with a given pathogen predisposes the individual host to secondary pathogens through immunosuppression [367,368]. In addition, pathogens of the same or similar geographical distribution, ecological tolerance, or sharing the same vectors are likely to co-infect the same individual host [369,370,371]. Antagonistic relationships between different pathogens within an individual host have also been reported. This usually happens through three mechanisms including host immune modulation, pathogen resources competition, or death of the host cell after the primary infection [365,366,372]. Nonetheless, co-circulation of pathogens within a host population and coinfections within an individual host are often detrimental due to additive pathology [373]. In addition, coinfection with closely related pathogens or causing diseases with overlapping clinical and pathological manifestation may hamper accurate diagnosis of the aetiology [256].

In areas with higher mosquito activities, WNV may be in co-circulation and co-infection with other flaviviruses, other arboviruses, other non-arboviruses, parasites, and bacteria as well. Coinfections of WNV with malaria have been document both in birds and human [374]. A study involving birds living in the suburban WNV hotspot areas of Chicago, USA, reported an inverse association between WNV and avian malaria [374]. This could potentially be attributed to the pathogenesis of both pathogens. WNV virus causes haemolytic anaemia in infected birds depriving plasmodia of their substrate [374]. Moreover, it has been suggested that WNV requires iron for replication, thus hampering the formation of erythrocytes needed to sustain *Plasmodium* in infected host [375]. In contrast, Baba et al. [376] reported 25% of human malaria (*P. falciparum*) cases in Nigeria in coinfection with WNV, suggesting a direct positive correlation between malaria and WNV infection. Host-associated difference in outcome of coinfection could potentially be due to the sequence of infection within the host. Therefore, it would be interesting to investigate the *Plasmodium*-WNV coinfection under controlled conditions. There has been one report of WNV coinfection with *Halicephalobus gingivalis* in a human, leading to a fatal meningoencephalitis. However, it remains unclear whether WNV infection preceded *Halicephalobus gingivalis* infection or the other way around [377].

There have been reports of natural coinfections of WNV with other arboviruses (flaviviruses and alphaviruses), including YFV, DENV, and chikungunya viruses (CHIKV) in humans [376]. Khan et al. [378] reported WNV and JEV coinfection in a 35-old patient with encephalitic clinical signs and symptoms. In WNV-endemic regions of Turkey, there have been cases of WNV and Toscana virus (TOSV) co-infections in two patients with severe febrile syndrome [379]. WNV and poxvirus coinfections have been reported among American crows (*Corvus brachyrhynchos*) in California [380]. Despite the severity of coinfections, fatal in many cases, it remains unclear whether WNV infection predisposes the host to secondary infection or if both infections occur independently of each other.

The role of coinfections in intermediate hosts and vectors of various diseases has been studied. Coinfection has also been studied in vectors and intermediate hosts of WNV. Several studies have explored the role of endosymbionts such as *Wolbachia*, salivary gland hypertrophy (SGH), and *Sodalis glossinidius* infection in mosquitoes and tsetse fly species in the control of human and animal infectious diseases such as DENV infection, CHIKV infection, and trypanosomiasis [381,382,383,384]. It was demonstrated that coinfection with *Wolbachia* reduces vector competence of *Aedes aegypti* in DENV and CHIKV transmission [381]. Dodson et al. [385] demonstrated that *Wolbachia* enhances WNV infection in *Cx. tarsalis* mosquitos. In contrast, Glaser and Meola [386] confirmed that *Wolbachia* impairs the vectorial competences of *Culex quinquefasciatus* in the transmission of WNV. Although symbiotic relationship might be used as mode of biological control of different diseases, different factors, including ecological, environmental, and public health, must be taken into account before using *Wolbachia* as a biological control of DENV in areas where both WNV and DENV are endemic.

Several studies have reported the antagonist relationship between WNV and insect-specific flaviviruses (ISFs). It has been reported that Palm Creek virus (PCV), an ISF discovered in Australia, significantly antagonises the replication of WNV *in vitro* with a reduction in titres of up to 43-fold [387]. Similarly, it was later demonstrated that PCV interferes with WNV replication once they both coinfected *Cx. annulirostris* mosquitoes [139]. Goenaga et al. [388] demonstrated the potential that infection of some WNV such as *Culex* spp. vectors with Nhumirim virus (NHUV) could compromise the efficient transmissibility of flaviviruses associated with human disease such as WNV. Furthermore, the flavivirus, Bamaga virus (BgV), also discovered in Australia, interferes with WNV replication *in vitro* and transmission *in vivo* [140]. However, BgV is not a “true” ISF, but sitting at the interface of both vertebrate infecting flaviviruses and ISFs [140,389]. All the other viruses share a common denominator; they all are restricted to mosquitoes. The mechanism of restriction to mosquito and suppression of WNV requires further investigation. Understanding these phenomena, might open some avenues for biological control of pathogenic flaviviruses such as WNV, ZIKV, YFV, and DENV.

## 7. West Nile Virus in Reptiles

WNV infection has been extensively studied in endotherms including humans, domestic and wild mammals, and birds under experimental or natural infection. There are a few reports of WNV infection in ectodermal animals such as reptiles. Except for a few epidemiological studies, most available reports are from experimental infections.

A sero-epidemiological study in Mexico found a WNV prevalence of 41% in the wild vs. 30% in captive Morelet’s crocodiles (*C. moreletii*) [22]. Another study by Farfan-Ale et al. [390] reported a prevalence of 86% in farmed crocodiles in Mexico. A prevalence as high as 70% was reported by Steinman et al. [27] in Nile crocodiles (*C. niloticus*) in Israel. All these reports were based on the screening of clinically healthy crocodiles. Several other serological surveys have been conducted and, WNV was detected in farmed Nile crocodiles in Israel [6], wild alligators in Florida [38], and free range alligators in Louisiana [6,39,391]. The first clinical disease due to WNV infection in crocodilian species was reported in farmed American alligators (*Alligator mississippiensis*) in the USA [179]. Following the first clinical case in 2001, there was a severe outbreak between 2001 and 2003 that caused the death of approximately 2000 alligators [179]. Similar clinical signs, including anorexia, weakness, swimming in circles, bloody diarrhea, and scoliosis, and lesions were seen in infected Nile crocodiles (*C. niloticus*) in Zambia. At post-mortem, lesions included pulmonary congestion, haemorrhagic intestines and trachea, and hydropericardium [344]. In contrast to American alligators and Nile crocodiles, WNV infection was diagnosed only in farmed, clinically healthy *C. porosus* in Australia [280]. Experimental studies suggested that American alligators and saltwater crocodiles are also WNV amplifiers [39,41].

In a WNV experimental infection of Common Garter Snakes (*Thamnophis sirtalis sirtalis*), approximately 56% became viraemic and seroconverted [305]. Some of the infected snakes produced a viremia of up to 10^5^ PFU/mL (enough to be infectious to mosquitoes), thus making these snakes competent amplifying hosts. Some infected individuals remained viraemic for up to 11 dpi. It was demonstrated that there was a direct correlation between the infectious dose and route of administration with viremia, seroconversion, and severity of the disease. The clinical features varied from subclinical to neurological signs [305]. Some experimentally infected snakes displayed a range of clinical forms varying from sudden death following an asymptomatic infection to the more common clinical signs such as aggression and paralysis, weakness, and cachexia secondary to inappetence. The lethargic state of severely sick animals subjected them to predation, thus potentially becoming the source of infection for their predators. At post-mortem of dead snakes, the virus was isolated from various organs including liver, kidney, heart, intestine, skin, and skeletal muscle. The main observed lesions were multifocal to coalescent histiocytic hepatitis and splenitis. Viral antigen was detected in the cytoplasm of the liver and spleen macrophages [305].

A study that consisted of seasonal sampling of six wild snake species in Pennsylvania, USA (Northern Ringneck Snake, *Diadophis punctatus punctatus*, Northern Water Snake, *Nerodia sipedon sipedon*, Midland Rat Snake, *Scotophis spiloides*, Northern Brown Snake, *Storeria dekayi dekayi*, Northern Redbelly Snake, *Storeria occipitomaculata occipitomaculata*, and Eastern Garter Snake, *Thamnophis sirtalis sirtalis*) reported a prevalence of 1.62% (2/123) by detection of viral RNA from oral swab samples. The positive snakes were all Eastern Garter Snake. This is another evidence of the potential role of wild reptiles in the life cycle and transmission to other hosts [23].

Experimentally infected green iguanas (*Iguana iguana*) seroconverted and virus-neutralising antibody titres were detected 28 dpi. The average virus titre in the green iguana was 10^3.2^ PFU/mL 4 dpi. The North American bullfrog (*Rana catesbeiana*) yielded a low but detectable viremia ranging between 10^1.9^ PFU/mL 1 dpi and 10^2.2^ PFU/mL at 3 dpi [391].

There have been a few attempts to study the pathogenesis of WNV in reptilians such as Common Garter Snake, American alligator, and saltwater crocodiles [38,39,41,279,288,391]. Alligators infected with WNV_NY99_ displayed neurological and gastrointestinal disease. During outbreaks, there is high morbidity in affected pens on farms. The mortality is generally low, but it can be as high as 60% in juvenile animals [288]. Virus titres in clinically ill animals were as high as 10^6.5^ PFU/mL in plasma, 10^8.4^ PFU/0.5 cm^3^ in heart, 10^8.9^ PFU/0.5 cm^3^ in liver and lung, 10^8.3^ PFU/0.5 cm^3^ in kidney and 10^6.6^ PFU/0.5 cm^3^ in the spinal cord and brain [38].

Primary skin lesions generally appeared 4–5 weeks following acute infection. However, the link between the cutaneous lymphohistiocytic proliferative syndrome, also known as “pix”, and WNV was only established and reported later [279,280,288,392]. “Pix” is defined as transluminant areas of about 1–2 mm on the crocodile skin or hide usually detected during skin processing. These lesions are commonly found on the ventral region extending from the ventral caudal part of the neck to the ventral pelvis [392].

Virus-associated lymphoid aggregates in less severe disease forms of WNV infection in crocodilians are found in the intestine, lung, stomach, skin, kidney, mesentery, oesophagus, eye, tonsil, brain, thyroid, ovary, conjunctiva, heart, testicle, pancreas, gall bladder, bone marrow, salivary gland, spinal cord, spleen, and ureter [279,288]. In addition to lymphoproliferative aggregates, other microscopic lesions include vacuolar changes in the adrenal gland and liver and rhabdomyolysis in muscles as a result of acute stress [288]. Lymphoid aggregates in various internal organs are also seen in saltwater crocodiles with “pix” skin lesions in the absence of any other pathologies and virus antigen or RNA has not so far been detected in these lymphoid aggregates, hence it is unknown whether they only reflect general immune activation in species without proper lymph nodes or the aggregates arise in foci previously infected with WNV [41].

Being cold-blooded, reptilians have a slow metabolism and lack lymphnodes, hence, a slow humoral immune response to WNV infection [393]. As a result, in some infected reptiles, it takes up to several weeks or even months for the antibodies to peak [41]. Likewise, it also takes time for the generated antibodies titres to decrease to baseline values [391]. WNV neutralising titres of as high as 1:640 were detected in American alligators 14 months post exposure to WNV infection [288]. Passive transfer of WNV-specific antibodies via the egg yolk has been demonstrated in saltwater crocodile hatchlings, which remain protected for up to 4 months or longer [41].

Despite some attempts to understand the pathogenesis of WNV infection in reptiles, particularly in crocodilians, the mechanisms of disease development have not been elucidated yet. Since WNV infection in *C. porosus* appears to have dramatically different outcomes compared to infection in American alligators, there is a need to investigate the infection in these apex predators under both natural and experimental settings. Such comparative studies may help elucidate immunological and immunopathological mechanisms and hence inform efforts to develop efficacious vaccines.

## 8. Diagnostic Approaches of WNV

Under clinical settings, the diagnosis of WNV infection is generally based on the clinical examination, laboratory testing and post-mortem examination. The clinical examination in humans is based on observation of clinical signs, including acute fever, anorexia and nausea, vomiting, eye pain, headache, myalgia, rash, lymphadenopathy, and arthralgia [394,395]. It has been demonstrated that multifocal chorioretinitis is a potential marker for severe WNV infection in humans. Thus, in addition to neurological examination, ophthalmological examination should be performed when WNV infection is suspected [396]. However, the limitation of the clinical diagnosis is that there is no pathognomonic clinical sign of the disease in any affected species. Moreover, in non-endemic areas, clinical examination is less reliable in animals, as the clinical presentation would suggest other infectious diseases with WNV not necessarily being on top of the differentials. In addition, the clinical diagnosis is in most cases presumptive; in humans, it should be supported by travel history. In areas where diseases of similar clinical manifestations are endemic such as malaria, WNV infection might not be on the list of differentials given similar clinical picture, epidemiological information, and mode of transmission.

The laboratory based diagnostic approaches comprise of virus isolation, RT-PCR, serology, and pathological examination. Serologically, the diagnosis is based on the detection of IgM and IgG antibodies against WNV. These antibodies can be detected 3–7 days post-exposure. Particularly, IgM can persistently be detected for up to two years, notably in horses, limiting their usefulness in a diagnostic context [397,398]. Blocking ELISA has been shown to be a reliable, cheap, and easy diagnostic tool in a laboratory setting. It consists of measuring the ability of the antibodies of the patient’s serum to inhibit the binding of monoclonal antibodies against NS1 and E protein epitopes [399,400]. This assay has the advantage of being versatile and species independent [401]. However, depending on the blocking antibody used in the ELISA, cross-reactivity with other flaviviruses may occur, as shown for MVEV, ALF and other locally circulating flaviviruses [402]. Nevertheless, since most vaccines candidates are developed based on prM and E protein, the NS1 targeted ELISA is often used to segregate post-infection antibodies from post-vaccinal antibodies [74,403]. However, the virus neutralising test (VNT) against WNV remains the gold standard test, as it has high specificity and not only detect the neutralising antibodies to the virus but also quantify the neutralising titres [402]. The limitation of this assay is that it takes a week to get the results and is relatively expensive; hence, its restricted use as a diagnostic tool.

Over the last decades, a nucleic acid-based molecular technique has been a rapid and most reliable diagnostic tool thanks to its sensitivity and specificity [404]. Currently, it is commonly used in the diagnosis of viral infections including WNV [405,406,407,408]. The most commonly used molecular diagnostic techniques include reverse transcription polymerase chain reaction (RT-PCR), quantitative RT-PCR (qRT-PCR) and in situ hybridisation [409]. qRT-PCR has an advantage over regular RT-PCR of quantifying the viral genome. The quantitation is achieved through monitoring the accumulation of double-strand DNA using DNA intercalating fluorescent dyes such as SYBR^®^ Green. Instead, the quantitation can be achieved through monitoring the amplification of specific target sequence using detection probes [410]. PCR primers should target NS5, the most conserved genome regions in nearly all flaviviruses [411]. The molecular diagnosis of WNV targets the E protein region, conserved across several WNV strains [412]. Being very sensitive, RT-PCR may detect the viral RNA from animals vaccinated with killed WNV vaccine [404]; therefore, while screening individuals vaccinated with such vaccines, PCR should be complemented with other diagnostic methods such as virus isolation. Alternatively, the RT-PCR should target WNV-NS5 to segregate viral RNA from the vaccine from replicating virus from infection. The testing should be done on serum or cerebrospinal fluid (CSF) samples.

The pathological diagnosis has always been useful in the clinical and experimental investigation of WNV infections. This diagnostic approach hinges on detection of macroscopic and microscopic lesions as well as the detection of viral antigen in histological tissue sections using IHC techniques [108,264,320,413,414,415]. IHC based on the NS1 or NS3 protein offers the most conclusive, definitive diagnosis as it confirms viral replication in tissues. However, IHC has some limitations, including the availability of specific antibodies, invasive methods of sample collection or otherwise, being strictly a post-mortem procedure. WNV NS1 has been shown to be the more specific and reliable diagnostic marker for acute phase WNV infection [105].

Shaikh et al. [416] developed an immunochromatographic strip assay known as RapidWN™ specific for WNV infection diagnosis for use in humans. This technique is based on the same principles as standard ELISA and it consists of detecting WNV IgM antibodies in plasma or serum from infected individuals. However, the method has the advantage over standard ELISA of being an in-house test, rapid, cost-effective, and user friendly. The test does not require a spacious laboratory with high tech equipment or highly trained personnel. Unfortunately, to date, there is no rapid test for veterinary use which could serve for early detection of WNV infection at a low cost. Therefore, this justifies the need for developing a user-friendly diagnostic tool for both medical and veterinary use.

## 9. Biomarkers of WNV Infection

WNV infection is generally associated with upregulation or, downregulation of expression of inflammatory cytokines and chemokines. Subjects with severe WNV infection have downregulated IL-4, IL-1β (peripheral blood cells), and CXCL1 (myeloid dendritic cells) [417,418]. There is other evidence of upregulation of IFN γ, TNFα an IL-6 in the CNS during the acute phase of neuroinvasive WNV infection [419]. Likewise, it was demonstrated that IL-10 gene expression is upregulated in plasma during the acute phase of infection with WNV [420]. However, IL-10 gene regulation is not WNV specific; thus, it would not serve for definitive diagnosis [421]. Other biomolecular markers have been suggested to be associated with the prognosis of WNV infection. For example, an increased brain actin cytoskeleton and Rho-family GTPases has been associated with neuroinvasive WNV infection. Furthermore, the upregulation of high mobility group box 1 (HMGB1) and peroxiredoxin (PRDX6) in brain has been associated with brain injury in infected mice. Moreover, glial fibrillary acidic protein (GFAP), expressed in mature astrocytes, has been found to be upregulated in the blood during the entire course of neuroinvasive disease. This finding suggests that blood GFAP levels could be a good candidate marker for brain damage during neuroinvasive WNV infection [422]. It is clear that a variety of molecule levels have been reported to be associated with various forms of WNV infection; thus, these molecules could potentially be relevant for prognosis investigation purposes.

## 10. WNV and One Health

WNV is an arbovirus that has a complex life cycle requiring an interaction of mosquito vectors, vertebrate reservoirs, and final hosts, which interact in a dynamic environment. Clinically, WNV infection occurs in both medical and veterinary settings. Climate conditions, particularly ambient temperature and rainfall, are fundamental drivers of mosquito abundance and amplification of WNV and infection dynamism in endemic areas [423].

The WNV infection is a transboundary disease of One Health (OH) concern [424]. There are several possibilities by which WNV could be introduced into nonendemic areas. These factors include transport of mosquitoes by ships, airplanes, or wind. Bird migration, bird trade, and human movements have also been proposed as possible ways WNV can spread [425]. The transboundary hypothesis was supported by evidence of a close relationship between WNV_NY99_ and WNV isolated from a dead goose in Israel in 1998 [153,426].

Studies have demonstrated the relationship between environment and the prevalence of WNV in humans as well as in animals. The alteration of natural habitat of WNV reservoirs and vectors by human activities such as land use, urbanization, and agriculture has led to increased prevalence of WNV in humans in endemic areas [427,428]. In Europe, there is a direct correlation between land irrigation and the increased incidence of WNV infection. Furthermore, rice fields, stagnant water, and wetland provide conducive environments for mosquito proliferation, resulting in WNV outbreaks [429,430]. Besides, the increased prevalence of WNV in horses was correlated with woodland and bushy areas that offer adequate nesting and resting to the WNV reservoirs and vectors [431].

Incidence of WNV infection in humans has been associated with patterns in temperature and precipitation, thus proving the seasonality of WNV [432,433]. Moreover, researchers have hypothesised that the USA WNV outbreak in 1999 was a result of mosquito booming following extreme summer temperatures and precipitation [51,153]. A similar scenario has been reported in the Danube Delta in Romania [434]. Furthermore, the eruption of the 2011 WNV_KUN_ infection in Australia was likely a result of an increased *Cx. annulirostris* population following an immense flooding event in Eastern Australia [211,329]. Generally, in wetlands, mosquito populations are driven by drought and rainfall alternation that subsequently impact the mosquito-borne infections [435].

Globalisation, travel, and trade have also been linked to introduction of WNV in non-endemic areas. All WNV infection cases reported in the United Kingdom are solely related to travel in endemic areas [424]. Lanciotti et al. [153] also hypothesised that travel of infected hosts (human or animals) could have contributed to the introduction of WNV in the USA. In addition to travel, there has been evidence of association of WNV incidence with people’s culture, traditions, and behaviour [436].

## 11. Control of WNV Infection and Disease

Like for most viral infection, there is no specific cure for WNV infection. Currently, the control of the disease consists of vaccination (in horse), vector control, and vaccine development for humans and other susceptible species.

### 11.1. Vaccination and Vaccine Development

There have been attempts to develop a vaccine against WNV in humans, but to date, there is no approved commercially available vaccine for use in human [437,438,439,440,441,442,443,444,445]. Progress has been made towards discovery of anti-WNV vaccine candidates with the main focus on DNA-delivered subunit or single-round infectious particles (SRIPs) [446,447] and chimeric vaccines [3,437,448,449,450].

Currently there are approved, commercially available vaccines for veterinary use; these include two formalin-inactivated vaccines and a DNA vaccine. One formalin inactivated WNV vaccine was developed by Innovator^®^, Fort Dodge, Princeton, NJ, USA. It was the first licensed WNV vaccine for veterinary use in the USA. The vaccine confers protective immunity to 94% of vaccinated horses. The other inactivated vaccine known as Vetera^®^ WNV is manufactured by Boehringer Ingelheim [451]. Although these vaccines are immunogenic, their main downside is that after the initial two injections administered 4 to 6 weeks apart, they both require a booster dose every six months to attain and maintain a fully protective response [452].

A recombinant, live attenuated vaccine is currently commercially available for veterinary use. The vaccine consists of a recombinant live canarypox virus that express the WNV prM and E transgenes derived from the WNV_NY99_ strain. The vaccine is commercially known as Recombitek^®^, Merial. The two initial doses are administered intramuscularly at a 5-week interval. After the first two doses, vaccinated horses are 100% protected against the disease for up to 1 year [445]. In vaccinated horses, neutralising antibodies were detected 26 days after the single dose [453]. The vaccine has also been successfully tested for use in dogs and cats [454].

Currently, several other vaccines against WNV in both humans and animals are at different developmental stages [438,439,442,449,450,455]. Among the vaccine candidates, three have reached the clinical trial phase. These include the live attenuated chimeric vaccine known as ChimeriVax-WNV vaccine. This vaccine uses the YFV-17D backbone to present the structural WNV prME genes. A similar chimeric vaccine is based on the DENV-4 backbone presenting structural WNV genes. The safety and efficacy of these two vaccines have been promising since they are well tolerated and induce decent neutralising titres [456]. The other vaccine candidate that has entered clinical trial is a non-infectious DNA construct, expressing WNV prME. The vaccine was safely administered in three doses [454,457].

Although all the flaviviruses are antigenically related [458,459], attempts to develop a single, cross-protecting vaccine have been unsuccessful. The concept of cross-neutralisation remains controversial. Although not fully proved, it is believed that cross-neutralisation is restricted to flaviviruses of the same sero-complex, i.e., more closely related viruses [459,460,461,462,463]. However, there is evidence of cross-protection where previous exposure to one flavivirus conferred protection against another flavivirus. One example is “partial” protection for WNV in individuals who live in DENV-endemic areas such as Latin America and the Caribbean [464,465]. Saron et al. [466] also demonstrated that vaccination against JEV may confer protection against secondary DENV-1 infection. This evidence is also supported by Li et al. [467]. Fox et al. [468] in their epidemiological study in Vietnam also demonstrated that vaccination against JEV led to a significant decrease of DENV infection.

With the current trend of globalisation and climate change, flavivirus infections have become not only of public health concern but also a global health issue. These threats need to be addressed using a one-health concept through the collaboration of different stakeholders, including medical doctors, (community) health workers, anthropologists [469], environmentalists, conservationists, veterinarians, and farmers, among others. In addition, the development of cross protecting vaccines would be a great success towards the control and potential eradication of pathogenic flavivirus infections. For example, the recent exploitation of the insect-specific Binjari virus provides a novel platform for the production of safe and efficient vaccines against a range of pathogenic flaviviruses using a rapid chimerisation technology [449,450].

### 11.2. Other Control Strategies

Without a cure or vaccine for WNV, controlling or reducing vector density, personal protection to reduce the risks of exposure and screening blood and organ donors remain the most effective ways to control WNV in endemic areas [470]. Biological control of flaviviruses including WNV has been explored. This is an indirect mode of control consisting of reducing vectors using different biological agents. The most commonly used agents are *Bacillus thuringiensis* serotype *israelensis* (Bti), a mosquito larvicide [471,472]. Considering the peri-domestic ecology of certain mosquito vectors, people should aim to destroy any breeding ground for mosquitoes. Furthermore, people should get rid of stagnant and standing water, and any equipment or areas where water may accumulate [315]. Pesticides have also been commonly used to control vectors of various disease vectors. The limitation for these two approaches is that they do not discriminate disease vectors from beneficial insects. Besides, pesticides may also negatively affect aquatic wildlife [473,474]. Although aerial pesticide spraying was demonstrated to be effective in the control of WNV infection in humans [474], the impact of pesticides on the environment should be collaboratively assessed by all the involved stakeholders. Infection modelling should be used to forecast outbreaks for preparedness and early response in case of outbreaks [475].

## 12. Conclusions

This review has highlighted the public health, One Health, and economic importance of WNV infection in various host species. With the current trend in climate change, the control of WNV should use a multidisciplinary integrated approach involving virologists, immunologists, medical professionals, veterinarians, epidemiologists, anthropologists, and environmentalists. As efforts are made to understand the pathogenesis of WNV in various animal species, including humans, proper use of land, and environmental conservation should aim to protect the natural habitat of both WNV reservoirs and vectors. Moreover, in view of damages caused by the most recent outbreaks around the world, there is an urgent need for effective and safe vaccines to control the disease in various species. Availability of effective and safe vaccines would control the disease without tampering with either WNV reservoirs or vectors, which also play various important roles in nature conservation and biodiversity.

## Figures and Tables

**Table 2:** Principal outbreaks of WNV infection in human and different animal species.

Country/Continent	Place/Region/City	Year	Host	Form of the Disease	WNV strain	Lineage	Reference
USA	New York City	1999	Human	Neuroinvasive	WNV_NY99_	Lineage 1	[153,228,301,319,320]
USA	New York	1999	Horse	Neuroinvasive	WNV_NY99_	Lineage 1	[319]
USA	Georgia	2001–2004	Birds	Neuroinvasive	WNV_NY99_	Lineage 1	[321,322,323,324]
USA	North America	2002	Birds	-	WNV_NY99_	Lineage 1	[325]
USA	Southern California	2004	Birds	Neuroinvasive and gastrointestinal	WNV_NY99_	Lineage 1	[326,327]
USA	District of Columbia California	2013	Horse	Neuroinvasive	WNV_NY99_	Lineage 1	[328]
Australia	New South Wales	2011	Horse	Neuroinvasive	WNVN_SW2011_	Lineage I	[329]
Romania	Several districts		Human	Neuroinvasive WNV Fever syndrome	Various strains of lineage 2	Lineage 2	[330]
Russia	Volgograd Region	1999	Human	Neuroinvasive		Lineage 1	[331]
Israel	Various geographical areas	2000	Human	Neuroinvasive Gastrointestinal			[287]
Serbia	-	2012	Human	Neuroinvasive WNV Fever syndrome Gastrointestinal	-	-	[2]
Romania	Several districts	2016–2017	Human	Neuroinvasive WNV Fever syndrome	WN-Romania-1996	Lineage 2	[306]
Greece	Various geographical areas	2010	Human	Neuroinvasive WNV Fever syndrome	Nea Santa-Greece-2010	Lineage 2	[150,332]
Hungary	Various geographical areas		Horse	Neuroinvasive	Various strains of lineage 2	Lineage 2	[333]
Italy	Various geographical areas		Horse Human	Neuroinvasive	Various strains of lineage 1 and 2	Lineage 1 and 2	[334]
Israel	Central Israel	2015	Human	Neuroinvasive WNV Fever syndrome	-	-	[335]
Israel	Eilat	1998	Birds	Neuroinvasive	-	lineage 1 and 2	[220]
Tunisia	Sfax area (South Eastern Tunisia)	1997	Human	Neuroinvasive	-	-	[336]
Democratic Republic of the Congo	Kisangani Kapalata military camp	1998	Humans	Neuroinvasive WNV Fever syndrome Gastrointestinal	-	-	[337]
Sudan	Nuba Mountains	2002	Human	Neuroinvasive	-	-	[338]
Tunisia	Monastir, Mahdia and Sousse	2003	Human	Neuroinvasive WNV Fever syndrome Gastrointestinal	-	-	[339]
South Africa	Pretoria	2008–2009	Human	Neuroinvasive	-	Lineage 1	[340]
Gabon	Libreville	2009	Human	Neuroinvasive	-	-	[341]
Madagascar		2010	Horse				[342]
South Africa	Ceres in the Western Cape	2010	Horse		SAE75/10	Lineage 1	[343]
Zambia	Southern Province	2019	Nile Crocodile	Neuroinvasive Cutaneous	Croc110/2019/ZM	Lineage 1a	[344]
